# 

**Published:** 1905-08

**Authors:** 


					﻿The Drug-Dni.turner’s. Story, g
I Vol. XXI. I
AUGUST, 1905.
>2,
I TE3C.A.SI
Medical Journal
; t Subscription, $ i .00 a Year, in Advance.
5 OF ♦ fCt®INGLK Copies, io Cents.
PUBLISHED AT AUSTIN, TEXAS, BY
F.IE. DANIEL., M. D.,
Proprietor.
PRINTED BY THE VON BOEOKM ANN-JONES CO.
Entered at the Postoffice at Austin, Texas, as Second-class Mail Matter.'
				

